# A multiscale theory for network advection- reaction-diffusion

**DOI:** 10.1007/s00285-026-02386-2

**Published:** 2026-04-09

**Authors:** Hadrien Oliveri, Emilia Cozzolino, Alain Goriely

**Affiliations:** 1 Max Planck Institute for Plant Breeding Research, Cologne, 50829 Germany; 2 Dipartimento di Matematica, Università di Roma Tor Vergata, Rome, 00133 Italy; 3 Mathematical Institute, University of Oxford, Oxford, OX2 6GG UK

**Keywords:** 92D30, 37N25, 34C60

## Abstract

Mathematical network models are extremely useful to capture complex propagation processes between different regions (nodes), e.g. the spread of an infectious agent between different countries, or the transport and replication of toxic proteins across different brain regions in neurodegenerative diseases. In these models, transport is modelled at the macroscale through an operator, the so-called *graph Laplacian*, based on the edge properties and topology, capturing the fluxes between different nodes of the network. However, this phenomenological approach fails to take into account the physical processes taking place, at the microscale, within the edge. A fundamental problem is then to obtain a transport operator from mechanistic principles based on the underlying transport process. Using advection-reaction-diffusion as a generic mechanism for inter-nodal exchanges, we derive a multiscale network transport model and derive the corresponding linear transport operator at the macroscale from first principles. This *effective* graph Laplacian is fully determined by the transport mechanisms along the edges at the microscale. We show that this operator correctly captures the transport, and we study its scaling properties with respect to edge length.

## Introduction

Systems where mass or information is transported between well-defined discrete regions connected by physical pathways can often be modelled as dynamical systems on a network. In these systems, a quantity is defined at a *node*, where it follows some local dynamics, and is carried to other nodes by *edges* through some form of physical transport. Applications range, among others, from mobility-driven epidemiology (Kuhl 2021) to protein trafficking across the brain connectome (Fornari et al. 2019, 2020; Brennan and Goriely 2025). While modelling the dynamics at the level of nodes is relatively straightforward, a central mathematical question is to specify the transport operator governing the nodal exchanges. In the simplest case, by analogy with the continuum case of diffusion, one can postulate that transport is modelled by a graph Laplacian built from the network topology and weighted by its physical properties such as edge length and diameter (Gautreau et al. 2007; Linka et al. 2020; Raj et al. 2012). However, there is no first principle dictating the particular form of the transport operator, or how it should be weighted, beside general physical requirements such as mass conservation, or the Fickian property that mass diffuses from high to low concentrations. Here, we look at this issue as a multiscale problem. Positing an actual physical mechanism of transport along the network edges at the microscale, we determine an *effective graph Laplacian* at the macroscale.

At the macroscale, we wish to obtain a generic network advection-reaction-diffusion system by combining i) transport between nodes through the edges; and ii) local reaction in the nodes (e.g. autocatalysis). The generic form of such a network model, for *N* nodes, is a system of *N* ordinary differential equations (w.r.t. time *t*) for the densities at the nodes, of the form (Kuhl 2021; Brennan and Goriely 2025)

1 $$\begin{aligned} \frac{\textrm{d}{}}{\textrm{d}{t}}\left( {\boldsymbol{\mathcal {V}} \boldsymbol{\rho }}\right) = \boldsymbol{\mathcal {V}} \boldsymbol{\mathcal {G}}(\boldsymbol{\rho }) + \textbf{L}\boldsymbol{\rho }. \end{aligned}$$

Here $\boldsymbol{\rho }=\left( {\rho _1,\dots ,\rho _N}\right)$ is the vector of densities (with unit mass per volume), expressing the densities of the considered species across all nodes of the network; and $\boldsymbol{\mathcal {V}} = {{\,\textrm{diag}\,}}\left( {\mathcal {V}_1,\dots ,\mathcal {V}_N}\right)$ is the diagonal matrix of nodal volumes, so that $\boldsymbol{\mathcal {V}} \boldsymbol{\rho }$ has the dimension of a mass. The vector function $\boldsymbol{\mathcal {G}}(\boldsymbol{\rho })$ captures the local nodal reaction, which may encompass various chemical reactions, as well as natural decay or clearance (Brennan et al. 2024; Ahern et al. 2025). Transport is assumed to be linear and encompasses various contributions, viz. passive Fickian diffusion, and active transport. Typically, these processes have been modelled through the so-called *graph Laplacian* $\textbf{L}$, built from the weighted adjacency matrix of the network (Putra et al. 2021).

This type of system is straightforward to analyse and solve numerically, and has proven highly effective in capturing key propagation mechanisms, as it can be readily validated and parameterised against data (Chaggar et al. 2025). However, from a physical perspective, a key issue is to obtain the appropriate form of both the reaction term $\boldsymbol{\mathcal {G}}$ and the transport operator $\textbf{L}$. Indeed, various authors have used different ad hoc conventions (Abdelnour et al. 2014; Raj et al. 2015; Pandya et al. 2017, 2019; Thompson et al. 2020)—some using Laplacian matrices that violate mass balance, as pointed out by Putra et al. (2021). Such inconsistency is rather unsettling, considering each model aspires to describe the same underlying physical processes of diffusion and transport.

The fundamental question being addressed here is then: given well-defined physical processes specifying reaction between species and transport along the edges (the microscale), can we derive a model—defined by both a reaction term and a transport operator in (1)—at the network scale (the macroscale), from first principles?

**Fig. 1:**
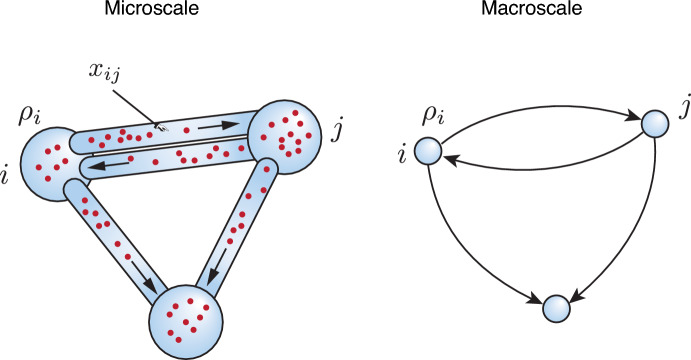
Schematic of the model’s structure. At the microscale, the system is a physical network where nodes are compartments with volumes $\mathcal {V}_i$ and the edges are (one-dimensional) tubes defining a continuum domain for the advection-reaction diffusion process (with orientation of the advection shown by the arrows). At the macroscale, the system is viewed as a mathematical network of nodes and edges where the nodal densities $\rho _i$ evolve according to the macroscopic governing equation (21).

To obtain the transport operator $\textbf{L}$ and the correct form of the reaction term, we must therefore start by considering the actual physical transport within each edge at the microscale. Here, we will assume a generic advection-reaction-diffusion transport on each edge. We will show that under suitable simplifying assumptions, we can solve the microscale problem on each edge for multiple species to obtain the change of density along the edges and match the fluxes at the end of the edges to those at each node. If the density profile along an edge depends on the nodal densities linearly, one can, in principle, obtain a linear operator, which we call the *effective graph Laplacian*, hence connecting the physics at the microscale to the macroscale behaviour (Fig. 1).

## General setup

A network of size *N* is defined as a pair $(\mathcal {V},\mathcal {E})$, where $\mathcal {V}=\{1,2,\dots ,N\}$ is the set of nodes and $\mathcal {E}\subseteq \mathcal {V}\times \mathcal {V}$ is the set of edges connecting them. Since active transport between nodes is generally not symmetric, the network is directed, with edge (*i*, *j*) distinct from edge (*j*, *i*). An edge (*i*, *j*) has arc length $\ell _{ij}$ and is assumed to have uniform cross-sectional area $w_{ij}$ so that the edge volume is given by $v_{ij}\approx \ell _{ij}w_{ij}$ (without summation on repeated indices). Note that, in the case where no physical edge (*i*, *j*) actually exists in the graph, we simply take $w_{ij}=0$ (so we consider $\mathcal E =\mathcal V\times \mathcal V$). We first consider the advection-diffusion-reaction of *n* species on a single edge before connecting the different edges to nodes.

## Advection-reaction-diffusion along an edge

At the microscale, we view a single edge as a slender one-dimensional tube with arclength coordinate $s\in \left[ 0,\ell \right]$ in which *n* species with density $\textbf{x} = \textbf{x}(s,t)\in \mathbb {R}^n$ interact through

2 $$\begin{aligned} \frac{\partial {\textbf{x}}}{\partial {t}} + \frac{\partial {\textbf{J}}}{\partial {s}} =\textbf{F}(\textbf{x}). \end{aligned}$$

Here, $\textbf{F}(\textbf{x})\in \mathbb {R}^n$ captures any source or sink, or chemical reaction between the species. The flux $\textbf{J}$ through the domain is given by

3 $$\begin{aligned} \textbf{J}= \textbf{V} \textbf{x} - \textbf{D} \frac{\partial {\textbf{x}}}{\partial {s}} , \end{aligned}$$

with $\textbf{V}$ the diagonal *transport velocity matrix*; and $\textbf{D}$ the positive-definite diagonal *matrix of diffusion coefficients*. Assuming that the dynamics at the nodes is much slower than that along the edges, and taking $\textbf{V}$ and $\textbf{D}$ to be constant in *s*, we consider the quasi-static problem expressed by

4 $$\begin{aligned} - \textbf{D} \textbf{x}''+\textbf{V} \textbf{x} ' =\textbf{F}(\textbf{x}) \end{aligned}$$

where the apostrophe denotes differentiation w.r.t. *s*. Here, we consider Dirichlet boundary conditions of the form

5 $$\begin{aligned} \textbf{x} (0) = \boldsymbol{\rho }_0(t), \quad \textbf{x} (\ell ) = \boldsymbol{\rho }_\ell (t) \end{aligned}$$

where the vectors $\boldsymbol{\rho }_0$ and $\boldsymbol{\rho }_\ell$ denote the (positive) densities at both ends of the domain. In a network, these will model the densities at the end nodes of a given edge, and will connect the solution of the advection-reaction-diffusion process at the edge scale to the node densities at the network scale. In general, when $\textbf{F}$ is nonlinear, there is no closed-form solution to (4) and one has to resort to numerical techniques. Here, we assume that the dominant behaviour at the microscale is governed by linear reaction and, accordingly, linearise the problem.

Assuming the densities remain small, we linearise the reaction term $\textbf{F}(\textbf{x}) = \textbf{f}_0 + \textbf{K} \textbf{x} + O(|\textbf{x}|^2 )$ and consider the simpler problem

6 $$\begin{aligned} - \textbf{D} \textbf{x}'' + \textbf{V} \textbf{x}' - \textbf{K} \textbf{x} = {\textbf{f}_0 }. \end{aligned}$$

Here $\textbf{V}$, $\textbf{D}$, $\textbf{K}$ and $\textbf{f}_0$ are constant in time (or slowly varying). They reflect different physical processes, thus are assumed to be independent. Hence, the problem (6, 5) is almost always non-singular and admits a unique solution. Note that, for a single species ($n=1$), if $K<0$ and $f_0 \ge 0$, the Hopf maximum principle for elliptic equations implies that $x(s)\ge 0$. The same result holds in the case of multiple species if $\textbf{K}$ is diagonal and ${{\,\textrm{diag}\,}}\left( {\textbf{K}}\right) <0$ entry-wise. In the general case of a non-diagonal $\textbf{K}$, the maximum principle for elliptic systems does not hold with the same generic conditions on the coefficients as in the case of elliptic equations (see, e.g., Pini 1953). Hence, the positivity result needs to be derived for particular classes of matrices $\textbf{K}$. For instance, if the system is cooperative, i.e. $K_{ij}\ge 0$ for $i \ne j$ and $\sum _{j}K_{ij}\le 0$ for all *i*, and has homogeneous Dirichlet boundary conditions, then Theorem 1.1 of De Figueiredo and Mitidieri (1990) guarantees positivity.

To solve (6), we observe that the system is equivalent to

7 $$\begin{aligned} \textbf{z}' = \textbf{Q} \textbf{z} - \textbf{q},\quad \text {with}\quad \textbf{z} = \left[ \begin{array}{c} \textbf{x} \\ \textbf{x}' \end{array} \right] , \quad \textbf{Q} = \left[ \begin{array}{cc} \textbf{0} & \textbf{I}_n \\ - \textbf{D}^{-1}\textbf{K} & \textbf{D}^{-1} \textbf{V} \end{array} \right] ,\quad \textbf{q} = \left[ \begin{array}{c} \textbf{0} \\ \textbf{D}^{-1}\textbf{f}_0 \end{array} \right] , \end{aligned}$$

with $\textbf{I}_n$ denoting the identity matrix of dimension $n\times n$. The general solution to this problem, for $\textbf{Q}$ non-singular, is of the form

8 $$\begin{aligned} \textbf{z}(s) = {\text {e}}^{\textbf{Q} s} \left( {\textbf{z}_0 - \textbf{Q}^{-1} \textbf{q}}\right) + \textbf{Q}^{-1} \textbf{q}, \end{aligned}$$

where $\textbf{z}_0 = \textbf{z}\left( {0}\right)$. Defining $\textbf{P} = \left[ \textbf{I}_n \,\, \textbf{0} \right]$ the $n\times 2n$ matrix which extracts the first *n* rows of a 2*n*-vector, we have

9 $$\begin{aligned} \boldsymbol{\rho }_0=\textbf{P} \textbf{z}_0, \quad \boldsymbol{\rho }_\ell = \textbf{P}\left( {{\text {e}}^{\textbf{Q}\ell } \textbf{z}_0 + (\textbf{I}_{2n} - {\text {e}}^{\textbf{Q}\ell } ) \textbf{Q}^{-1} \textbf{q} }\right) . \end{aligned}$$

These equations together form a linear system for $\textbf{z}_{0}$:

10a $$\begin{aligned}&\textbf{M} \textbf{z}_0 = \textbf{k} , \quad \textbf{k} = \left[ \begin{array}{c} \boldsymbol{\rho }_0 \\ \boldsymbol{\rho }_\ell + \textbf{P} ({\text {e}}^{\textbf{Q}\ell }-\textbf{I}_{2n} ) \textbf{Q}^{-1} \textbf{q}\end{array} \right] ,\nonumber \\&\quad \textbf{M} = \left[ \begin{array}{cc} \textbf{P} \\ \textbf{P} {\text {e}}^{\textbf{Q}\ell }\end{array} \right] = {\begin{bmatrix} \textbf{I}_n & \textbf{0}\\ {\left[ \textrm{e}^{\textbf{Q} \ell } \right] _{1:n,:}} \end{bmatrix}}\in \mathbb {R}^{2n\times 2n}, \end{aligned}$$

where the subscript ‘1 : *n*,  : ’ denotes extraction of the first *n* rows of the $2n\times 2n$ matrix $\textrm{e}^{\textbf{Q} \ell }$. If $\textbf{M}$ is non-singular, we obtain $\textbf{z}_0$ as a function of $\boldsymbol{\rho }_0$ and $\boldsymbol{\rho }_1$ as $\textbf{z}_0 = \textbf{M}^{-1} \textbf{k}$. Replacing this result in (8), the solution for $\textbf{z}(s)$ is given by

11 $$\begin{aligned} \textbf{z}(s) = {\text {e}}^{\textbf{Q} s} \left( {\textbf{M}^{-1} \textbf{k} - \textbf{Q}^{-1} \textbf{q}}\right) + \textbf{Q}^{-1} \textbf{q}. \end{aligned}$$

From this solution, we obtain the fluxes (3)

12a $$\begin{aligned} &  \textbf{J}^- {:}{=} \textbf{J} (0) = \left( {\textbf{V} \textbf{P} - \textbf{D} \textbf{P} \textbf{Q} }\right) \textbf{M}^{-1} \textbf{k} + \textbf{f}_0, \end{aligned}$$

12b $$\begin{aligned} &  \textbf{J}^+ {:}{=} \textbf{J} (\ell ) = \left( {\textbf{V} \textbf{P} - \textbf{D} \textbf{P} \textbf{Q} }\right) {\text {e}}^{\textbf{Q}\ell } \textbf{M}^{-1} \textbf{k} + \left( {\textbf{V} \textbf{P} (\textbf{I}_{2n} - {\text {e}}^{\textbf{Q}\ell } ) + \textbf{D} \textbf{P} \textbf{Q}{\text {e}}^{\textbf{Q}\ell }}\right) \textbf{Q}^{-1} \textbf{q} . \end{aligned}$$

Note that solvability under given Dirichlet conditions, as well as the positivity of the resulting solution, are not guaranteed a priori. Indeed, the determinant of $\textbf{M}$ may vanish for particular parameter values. For edge lengths $\ell$ exceeding a threshold dictated by these values, the solution will become non-positive and thus unphysical. In Sects. 5, 6, we discuss this issue in more detail for the one-species case. For two species, a solvability condition can be expressed in terms of the roots of three fourth-degree polynomials (not shown). While deriving an explicit solvability condition for $n>1$ is hopeless, a numerical check is feasible and easily implemented for given parameters.

To summarize, from the densities at both ends of the edge, we obtain the density profile and fluxes along that edge, in the steady, linearized regime. We can now extend this solution to the whole network and describe the transport of material between nodes.

## Network model

We now turn to the case of a full network made of nodes and edges which transport species between those nodes. Here the strategy is to use the solution obtained in Sect. 3 to express the fluxes between the nodes and thereby derive the effective Laplacian operator.

### Graph advection-reaction-diffusion and effective Laplacian

We describe the dynamics of evolution of the chemical species on the graph. Let $x_{ij}^\mu (s)$ be the edge density of species $\mu$ on edge $\left( {i,j}\right)$ at position $s\in \left[ 0,\ell _{ij} \right]$. The node density at node *i* for the species $\mu$ is noted $\rho _i^\mu$. The advection-reaction-diffusion equations on the edge are then coupled with Dirichlet data by requiring that $x_{ij}^\mu$ is continuous across the edge-node interface

13 $$\begin{aligned} x_{ij}^\mu (0) = \rho _i^\mu , \quad x_{ij}^\mu (\ell _{ij}) = \rho _j^\mu , \end{aligned}$$

and using the explicit quasi-static solution (8). Thus, for any given set of node densities $\rho _i^\mu$, the density profiles along the edges are fully determined.

As a starting point we look at the balance of mass at each node and each edge. The mass of species $\mu$ carried by edge $(i,j)\in \mathcal {E}$ is

14 $$\begin{aligned} m_{ij}^\mu = \int _0^{\ell _{ij}} w_{ij} x_{ij}^\mu (s)\, \textrm{d}{s} . \end{aligned}$$

In our context, given the quasi-static solution (11) for $x_{ij}^\mu$, we have that the mass carried by the edge is a function of the various densities in the associated nodes, $m_{ij}^\mu = m_{ij}^\mu (\rho _i^1, \dots , \rho _i^n,\rho _j^1, \dots , \rho _j^n)$. Since the source term $F^\mu$ along the edge (noted $F_{ij}^\mu$ for this edge) does not depend explicitly on time, direct time differentiation provides

15 $$\begin{aligned} \dot{m}_{ij}^\mu = \sum _{\nu =1}^n \frac{\partial {m_{ij}^\mu }}{\partial {\rho _i^\nu }} \dot{\rho }_i^\nu + \frac{\partial {m_{ij}^\mu }}{\partial {\rho _j^\nu }} \dot{\rho }_j^\nu . \end{aligned}$$

The mass intake at each node *i* encompasses three types of terms: i) a bulk source ($\mathcal {G}_i^\mu$) accounting, e.g., for local clearance, sources and other chemical reactions (local node reactions); ii) the flux terms $(J^\pm )^\mu _{ij}$ due to exchanges with other nodes (node-node fluxes); and iii) an additional flux corresponding to a net transfer from the neighbouring edge (edge-node fluxes); cf. Tora et al. (2025); Bertsch et al. (2025). In total we write

16 $$\begin{aligned} \frac{\textrm{d}{}}{\textrm{d}{t}}\left( {\mathcal {V}_i \rho _i^\mu }\right)&= \underbrace{\mathcal {V}_i \mathcal {G}_i^\mu \left( {\rho _i^1, \dots , \rho _i^n}\right) }_{\text {Local nodal reactions}} + \underbrace{\left( {\sum _{j = 1 }^N w_{ji} (J^+)_{ji}^\mu - w_{ij} (J^-)_{ij}^\mu }\right) }_{\text {Node-node fluxes}}\nonumber \\&\quad - \underbrace{\left( {\dot{\rho }_i^\mu \sum _{j=1}^N \sum _{\nu =1}^n\left( {\frac{\partial {m_{ij}^\nu }}{\partial {\rho _i^\mu }} + \frac{\partial {m_{ji}^\nu }}{\partial {\rho _i^\mu }} }\right) }\right) }_{\text {Edge-node fluxes}}. \end{aligned}$$

The last term in (16) can be interpreted as a correction which accounts for the mass transfer from the node back into the edges due to the change of the Dirichlet condition. Indeed, the latter results in a change of the quasi-static solution for the edges and thus in the mass (14) carried by each edge. Thus the total mass carried by the edges varies with the boundary conditions, and this term models the corresponding change in mass at the node so that the total mass of the system is conserved (cf. Sect. 4.2). The key modelling assumption is the effective separability of the variation of the mass carried by the single edge (*i*, *j*) between nodes *i* and *j*, namely the separation of the contributions $\left( {{\partial {m_{ij}^\nu }}/{\partial {\rho _i^\mu }}}\right) \dot{\rho }_i^\nu$ for *i* and $\left( {{\partial {m_{ij}^\nu }}/{\partial {\rho _j^\mu }}}\right) \dot{\rho }_j^\nu$ for *j*. In other words, this means that the mass intake in the edge (*i*, *j*) compensating for a change $\dot{\rho }_i^\mu$ of the boundary condition at node *i* comes only from node *i* itself (and not from *j*).

In virtue of the quasi-static solutions for the edge concentrations, (8, 12), the $m_{ij}^\mu$ and $(J^\pm )_{ij}^\mu$ can be written as explicit functions of the $\rho _i^\mu$. Thus, the $N\times n$ equations (16) form a closed system for the $\rho _i^\mu$. The goal now is to rewrite these equations in a canonical form revealing the structure of the effective graph Laplacian.

From (12), we see that the left and right fluxes—respectively $(J^-)_{ij}^\mu$ and $(J^+)_{ij}^\mu$—are affine functions of the $\rho _i^\mu$ and $\rho _j^\mu$, that is, they can both be written in the form

17 $$\begin{aligned} w_{ij} (J^\pm )_{ij}^\mu = (\omega ^\pm )_{ij}^\mu + \sum _{\nu = 1 }^n (\mathbb {A}^\pm )_{ij}^{\mu \nu } \rho _i^\nu +(\mathbb {B}^\pm )_{ij}^{\mu \nu } \rho _j^\nu . \end{aligned}$$

Explicit expressions for the constant coefficients $(\omega ^\pm )_{ij}^\mu$, $(\mathbb {A}^\pm )_{ij}^{\mu \nu }$ and $(\mathbb {B}^\pm )_{ij}^{\mu \nu }$ are straightforward to derive from (10a, 12), however such expressions are not required to establish important properties of the Laplacian such as mass conservation, thus we omit them at this stage (explicit expressions will be considered in (Sect. 5). The expression (17) thus allows us to rewrite the second term in the r.h.s. of (16) as

18 $$\begin{aligned} \sum _{j = 1 }^N w_{ji} (J^+)_{ji}^\mu - w_{ij} (J^-)_{ij}^\mu =\Omega _i^\mu + \sum _{j=1}^N\sum _{\nu = 1 }^n\mathbb {L}_{ij}^{\mu \nu } \rho _j^\nu , \end{aligned}$$

with the *effective Laplacian tensor* $\mathbb {L}$ and *base source* $\mathbf {\Omega }$ given by

19a $$\begin{aligned} \mathbf {\mathbb {L}}_{ij}^{\mu \nu } = (\mathbb {A}^+)_{ji}^{\mu \nu } - (\mathbf {\mathbb {B}}^-)_{ij}^{\mu \nu } - \delta _{ij}\sum _{k = 1 }^N \left[ (\mathbb {A}^-)_{ik}^{\mu \nu } - (\mathbb {B}^+)_{ki}^{\mu \nu } \right] ; \end{aligned}$$

19b $$\begin{aligned} \Omega _i^\mu = \sum _{j = 1 }^N(\omega ^+)_{ji}^\mu - (\omega ^-)_{ij}^\mu . \end{aligned}$$

By the same linearity argument, we can rewrite (14) as

20 $$\begin{aligned} m_{ij}^\mu = r^\mu _{ij}+\sum _{\nu }^n\mathbb {M}_{ij}^{\mu \nu } \rho _i^{\nu } + \mathbb {N}_{ij}^{\mu \nu } \rho _j^{\nu } . \end{aligned}$$

Finally, using (18)–(20), assuming that the $\mathbb {M}_{ij}^{\mu \nu }$ and $\mathbb {N}_{ij}^{\mu \nu }$ vary slowly in the slow timescale of the network so that we can neglect $\dot{\mathbb {M}}_{ij}^{\mu \nu }\rho _i^\nu$ and $\dot{\mathbb {N}}_{ij}^{\mu \nu }\rho _i^\nu$, we rewrite (16) as

21 $$\begin{aligned} \frac{\textrm{d}{}}{\textrm{d}{t}}\left( {\mathcal {\hat{V}}_i^\mu \rho _i^\mu }\right) = \mathcal {\hat{V}}_i^\mu \hat{\mathcal {G}}_i^\mu (\rho _i^1, \dots , \rho _i^n)+ \sum _{j=1}^N\sum _{\nu = 1 }^n \mathbb {L}_{ij}^{\mu \nu } \rho _j^\nu ,\quad \forall \left( {i,\mu }\right) \in \left[ 1,N \right] \times \left[ 1,n \right] , \end{aligned}$$

providing the canonical form of the system which extends (1) to the case of *n* species. Here we have introduced the hatted quantities

22a $$\begin{aligned} \mathcal {\hat{V}}_i^\mu {:}{=}\mathcal {V}_i + \sum _{j=1}^N\sum _{\nu = 1}^n \left( {\mathbb {M}^{\nu \mu }_{ij} + \mathbb {N}^{\nu \mu }_{ji}}\right) , \end{aligned}$$

22b $$\begin{aligned} \mathcal {\hat{V}}_i ^\mu \hat{\mathcal {G}}_i^\mu \left( {\rho _i^1, \dots , \rho _i^n}\right) {:}{=}\mathcal {V}_i \mathcal {G}_i^\mu \left( {\rho _i^1, \dots , \rho _i^n}\right) + \Omega _i^\mu , \end{aligned}$$

which define respectively the *effective volume matrix* and the *effective nodal source matrix*. Note that the terms in the r.h.s. of (22a) are separate physical quantities defined independently, thus, the $\mathcal {\hat{V}}_i^\mu$ are non-zero in general and the problem is non-degenerate. In the case of a single species, we can recover the standard form (1) by substituting the volume and source matrices $\boldsymbol{\mathcal {V}}$ and $\boldsymbol{\mathcal {G}}$ with their effective, corrected counterpart.

Finally, we observe from (14) that if the edges are short, the load $m_{ij}^\mu$ carried by the edges should scale as $\sim w_{ij}\ell _{ij}$, i.e. like the volume of the edges. Thus, since $\mathbb {M}_{ij}^{\nu \mu }={\partial {m_{ij}^\nu }}/{\partial {\rho _i^\mu }}$ and $\mathbb {N}_{ij}^{\nu \mu }={\partial {m_{ij}^\nu }}/{\partial {\rho _j^\mu }}$, when the nodal volumes are large compared to the edge volumes, we can neglect the sum in (22a) and take $\mathcal {\hat{V}}_i^\mu = \mathcal {V}_i$.

**Fig. 2:**
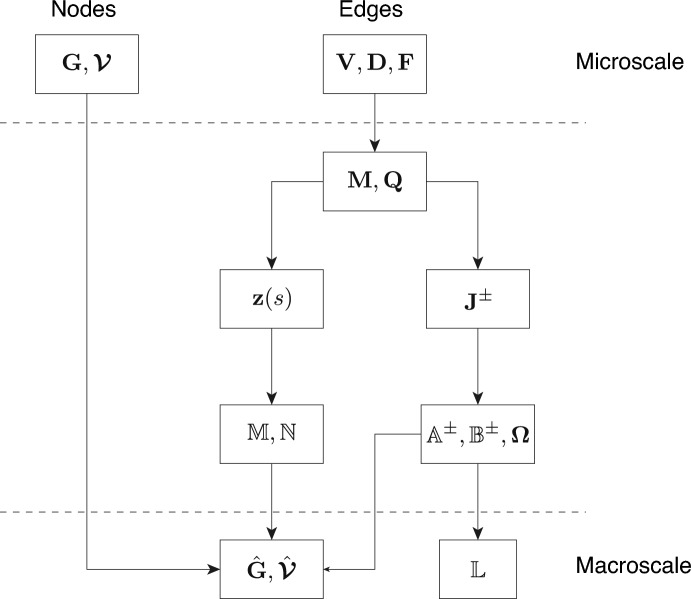
A summary of the upscaling process. From the initial microscale data provided by nodal and edge processes, we obtain the macroscale transport, reaction, and volume terms at the macroscale.

Since the operators introduced in this section are quite involved, we summarize the upscaling process in Fig. 2: we started with information at the microscale concerning edge and nodal processes. Then through upscaling, we obtain at the macroscale the different operators for the evolution of the nodal concentrations. These operators are explicitly given by the properties of the system at the microscale.

### Mass conservation property of the effective Laplacian

In this section, we establish properties of the Laplacian associated with mass conservation, a key requirement for a physical transport operator.

#### General case

The Laplacian tensor verifies the $N\times n$ mass conservation property (for all $j \in \mathcal {V}$ and $\nu \in \left[ 1,\dots , n \right]$)

23 $$\begin{aligned} \sum _{\mu =1}^n \sum _{i=1}^N {\mathbb {L}_{ij}^{\mu \nu }- \mathbb {A}_{ji}^{\mu \nu } - \mathbb {B}_{ij}^{\mu \nu }} = 0. \end{aligned}$$

with $\mathbb {A}_{ij}^{\mu \nu } {:}{=}(\mathbb {A}^+)_{ij}^{\mu \nu } - (\mathbb {A}^-)_{ij}^{\mu \nu }$ and $\mathbb {B}_{ij}^{\mu \nu } {:}{=}(\mathbb {B}^+)_{ij}^{\mu \nu } - (\mathbb {B}^-)_{ij}^{\mu \nu }$ capturing the mass production along the edges. This relation extends the mass conservation criterion of Putra et al. (2021) to multiple species and including sources along the edges. Specifically, the corrected Laplacian $\widehat{\mathbb {L}}_{ij}^{\mu \nu } {:}{=}\mathbb {L}_{ij}^{\mu \nu }- \mathbb {A}_{ji}^{\mu \nu } - \mathbb {B}_{ij}^{\mu \nu }$ is mass conserving, and is obtained by removing the additional contributions of non-mass-conserving reactions along the edges.

The mass conservation property (23) can be established from direct calculation using (19a). To see the link between (23) and mass evolution, we first define the total mass carried by the nodes and the edges, respectively as

24 $$\begin{aligned} M_{V} {:}{=}\sum _{\mu =1}^n\sum _{i=1}^N \mathcal {V}_i\rho _i^\mu ,\quad M_{E} {:}{=}\sum _{\mu =1}^n \sum _{i=1}^N\sum _{j=1}^N m_{ij}^\mu , \end{aligned}$$

where, we recall, $$m_{ij}^\mu $$ denotes the mass of species $$\mu $$ carried by the edge (*i*, *j*) as given by (14). In total, we define the total load of the network as $$ M = M_{V} + M_{E}$$. By summing the $$n\times N$$ equations (16) over the nodes and species types we write

25a $$\begin{aligned} \dot{M}_{V}&=\sum _{\mu =1}^n\sum _{i=1}^N\left( \mathcal {V}_i \mathcal {G}_i^\mu \left( {\rho _i^1, \dots , \rho _i^n}\right) + \sum _{j = 1 }^N w_{ji} (J^+)_{ji}^\mu - w_{ij} (J^-)_{ij}^\mu \right. \nonumber \\&\qquad \left. - \sum _{j = 1 }^N \sum _{\nu =1}^n \left( {\frac{\partial {m_{ij}^\nu }}{\partial {\rho _i^\mu }} + \frac{\partial {m_{ji}^\nu }}{\partial {\rho _i^\mu }} }\right) \dot{\rho }_i^\mu \right) , \end{aligned}$$

25b $$\begin{aligned} \dot{M}_{E} =\sum _{\mu =1}^n\sum _{i=1}^N\sum _{j=1}^N \sum _{\nu =1}^n \frac{\partial {m_{ij}^\mu }}{\partial {\rho _i^\nu }} \dot{\rho }_i^\nu + \frac{\partial {m_{ij}^\mu }}{\partial {\rho _j^\nu }} \dot{\rho }_j^\nu =\sum _{\mu =1}^n\sum _{i=1}^N\sum _{j=1}^N \sum _{\nu =1}^n \left( {\frac{\partial {m_{ij}^\nu }}{\partial {\rho _i^\mu }} + \frac{\partial {m_{ji}^\nu }}{\partial {\rho _j^\mu }} }\right) \dot{\rho }_i^\mu . \end{aligned}$$

Summing the two contributions we derive

26 $$\begin{aligned} \dot{M} =\sum _{\mu =1}^n\sum _{i=1}^N\left( {\mathcal {V}_i \mathcal {G}_i^\mu \left( {\rho _i^1, \dots , \rho _i^n}\right) + \sum _{j = 1 }^N w_{ji} (J^+)_{ji}^\mu - w_{ij} (J^-)_{ij}^\mu }\right) . \end{aligned}$$

We now examine the r.h.s. which captures the transport. We start by observing that the net mass *produced* within an edge can be expressed using (4, 17) as

27 $$\begin{aligned} w_{ij} (J^+)_{ij}^\mu - w_{ij} (J^-)_{ij}^\mu = \int _0^{\ell _{ij}} w_{ij}F_{ij}^\mu (x_{ij}^1,\dots ,x_{ij}^n) \approx \omega _{ij}^\mu + \sum _{\nu =1}^n \mathbb {A}_{ij}^{\mu \nu } \rho _i^\nu + \mathbb {B}_{ij}^{\mu \nu } \rho _j^\nu , \end{aligned}$$

with $\omega _{ij}^\mu {:}{=}(\omega ^+)_{ij}^\mu - (\omega ^-)_{ij}^\mu$ (the approximation sign expresses that the function $F^\mu _{ij}$ is here linearised w.r.t. the $x_{ij}^\mu$, as detailed in Sect. 3). Thus, summing over all edges and species, we can rearrange the terms in the last sum in (26), then use (27) to write:

28 $$\begin{aligned} \sum _{\mu =1}^n\sum _{i=1}^N\sum _{j = 1}^N {w_{ji} (J^+)_{ji}^\mu - w_{ij} (J^-)_{ij}^\mu }&= \sum _{\mu =1}^n\sum _{i=1}^N\sum _{j = 1}^N {w_{ij} (J^+)_{ij}^\mu - w_{ij} (J^-)_{ij}^\mu } \nonumber \\&\approx \sum _{\mu =1}^n\sum _{i=1}^N\sum _{j = 1 }^N \left( {\omega _{ij}^\mu + \sum _{\nu =1}^n \mathbb {A}_{ij}^{\mu \nu } \rho _i^\nu + \mathbb {B}_{ij}^{\mu \nu } \rho _j^\nu }\right) . \end{aligned}$$

Noting, from basic manipulation rules for sums, that

29 $$\begin{aligned} \sum _{\mu =1}^n\sum _{i=1}^N\sum _{j = 1 }^N \sum _{\nu =1}^n&\left( {\mathbb {A}_{ij}^{\mu \nu } \rho _i^\nu + \mathbb {B}_{ij}^{\mu \nu } \rho _j^\nu }\right) = \sum _{j = 1 }^N \sum _{\nu =1}^n \left( {\sum _{\mu =1}^n\sum _{i=1}^N \left( {\mathbb {A}_{ji}^{\mu \nu } + \mathbb {B}_{ij}^{\mu \nu } }\right) }\right) \rho _j^\nu , \end{aligned}$$

then using (18, 28, 29) and rearranging the terms, we obtain the relation

30 $$\begin{aligned} \sum _{j=1}^N\sum _{\nu = 1 }^n \left( {\sum _{\mu =1}^n \sum _{i=1}^N \left( {\mathbb {L}_{ij}^{\mu \nu }- \mathbb {A}_{ji}^{\mu \nu } - \mathbb {B}_{ij}^{\mu \nu }}\right) }\right) \rho _j^\nu = \sum _{\mu =1}^n\sum _{i=1}^N\left( {- \Omega _i^\mu + \sum _{j=1}^N \omega _{ij}^\mu }\right) . \end{aligned}$$

From the definition of the $\Omega _i^\mu$ (19b) we see directly that the r.h.s. actually vanishes:

31 $$\begin{aligned} \sum _{\mu =1}^n\sum _{i=1}^N\left( {- \Omega _i^\mu + \sum _{j=1}^N \omega _{ij}^\mu }\right) =0. \end{aligned}$$

Finally, since the $\rho _i^\mu$ are arbitrary in (30), the $n\times N$ mass conservation properties (23) must hold, which proves the mass conservation property.

#### Mass-conserving reaction

An interesting case is

32 $$\begin{aligned} \sum _{\mu =1}^n F_{ij}^\mu = 0 , \quad \forall (i,j)\in \mathcal {E}, \end{aligned}$$

corresponding to a scenario where species react in a way that conserves the total mass. This is the case of closed systems, including cyclic reactions (e.g. $A\rightarrow B$, $B\rightarrow C$ and $C\rightarrow A$), or contagion processes (e.g. $S+I\rightarrow I+I$). Here we show that

33 $$\begin{aligned} \sum _{\mu =1}^n \omega _{ij}^\mu = 0,\quad \sum _{\mu =1}^n \mathbb {A}_{ij}^{\mu \nu }= 0, \quad \sum _{\mu =1}^n \mathbb {B}_{ij}^{\mu \nu } =0, \end{aligned}$$

which directly yields, from (23),

34 $$\begin{aligned} \sum _{i=1}^N\sum _{\mu =1}^n \mathbb {L}_{ij}^{\mu \nu } = 0. \end{aligned}$$

#### Fully decoupled system

In the case where the advection-reaction-diffusion system is fully decoupled, we have that $\mathbb {L}_{ij}^{\mu \nu }=\mathbb {A}_{ij}^{\mu \nu }=\mathbb {B}_{ij}^{\mu \nu }=0$ if $\mu \ne \nu$, thus for all *j* and $\mu$:

35 $$\begin{aligned} \sum _{i=1}^N {\mathbb {L}_{ij}^{\mu \mu }- \mathbb {A}_{ji}^{\mu \mu } - \mathbb {B}_{ij}^{\mu \mu }} = 0. \end{aligned}$$

In particular for a closed system of one species with Laplacian $\textbf{L}$ and for which $F_{ij}=0$, the mass balance property reduces to the relation $\textbf{1}_N \textbf{L} = \textbf{0}$, with $\textbf{1}_N {:}{=}\left( {1,\dots ,1}\right) \in \mathbb {R}^N$ (Putra et al. 2021).

### Fick’s fixed point property

Finally, we verify that the graph Laplacian obeys the Fickian property that, in the sole presence of diffusion (i.e. in the absence of advection, conversion and sources/sinks), the uniform state $\rho _i^\mu \equiv 1$ is a fixed point. For this we require that for all *i* and $\mu$ (21)

36 $$\begin{aligned} \sum _{j=1}^N\sum _{\nu = 1 }^n \mathbb {L}_{ij}^{\mu \nu } =0. \end{aligned}$$

Since the system here is decoupled, this expression simplifies to (35)

37 $$\begin{aligned} \sum _{j=1}^N \mathbb {L}_{ij}^{\mu \mu } =0. \end{aligned}$$

The reaction is mass-conserving, thus we also have the equality (34)

38 $$\begin{aligned} \sum _{j=1}^N \mathbb {L}_{ji}^{\mu \mu } = 0. \end{aligned}$$

Here the Laplacian is symmetric and the two previous conditions are then equivalent (Putra et al. 2021), which proves the Fickian property.

## The case of one species

### General case

We henceforth examine the case of a single species ($n=1$) diffusing, reacting and being advected on the network. In this case, the linearised advection-reaction-diffusion equation (6) for one edge reduces to

39 $$\begin{aligned} -Dx''+Vx'-Kx = f_0. \end{aligned}$$

Using the explicit form of the determinant of $\textbf{M}$,

40 $$\begin{aligned} \det \textbf{M} = \frac{2 D }{\sqrt{V^2-4 DK}}\exp \left( {\frac{\ell V}{2 D}}\right) \sinh \left( \frac{\ell \sqrt{V^2-4 DK}}{2 D}\right) , \end{aligned}$$

we establish that this system always has a solution unless

41 $$\begin{aligned} \frac{\ell \sqrt{4 DK-V^2}}{2\pi D} \in \mathbb {N}^*. \end{aligned}$$

In particular, in the advection-dominated regime $V^2 > 4DK$, the square root is imaginary and solvability is always guaranteed. This loss of solvability at discrete values is not surprising and is also found in other linear systems subject to Dirichlet constraints (consider, e.g., the impossible scenario of a pendulum required to be in two different positions before and after a full-period swing). In our case, we shall see that this constraint results in a limitation on the maximum edge length. More precisely, we will assume $\ell \ll 2\pi D / \sqrt{4DK-V^2}$ whenever $4DK>V^2$ (cf. Sect. 6 for an exemplar case).

Adapting the results of Sect. 4.1 to the case of one species, the graph Laplacian is built from the eight constant matrices $$A_{ij}^-$$, $$A_{ij}^+$$, $$ B_{ij}^-$$, $$B_{ij}^-$$, $$\omega _{ij}^+$$, $$\omega _{ij}^+$$, $$M_{ij}$$ and $$N_{ij}$$ given by

42a $$\begin{aligned} A_{ij}^- = \frac{w_{ij}}{2} \left[ V_{ij}+\sqrt{\Delta _{ij}} \coth \left( \frac{\ell _{ij}\sqrt{\Delta _{ij} } }{2 D_{ij}}\right) \right] ; \end{aligned}$$

42b $$\begin{aligned} A_{ij}^+ = \frac{w_{ij} \sqrt{\Delta _{ij}}}{2} \exp \left( {\frac{\ell _{ij} V_{ij}}{2 D_{ij}}}\right) {{\,\textrm{csch}\,}}\left( \frac{\ell _{ij}\sqrt{\Delta _{ij}}}{2 D_{ij}}\right) \end{aligned}$$

42c $$\begin{aligned} B_{ij}^-= -\frac{w_{ij}\sqrt{\Delta _{ij} }}{2} \exp \left( {-\frac{\ell _{ij} V_{ij}}{2 D_{ij}}}\right) {{\,\textrm{csch}\,}}\left( \frac{\ell _{ij}\sqrt{\Delta _{ij} } }{2 D_{ij}}\right) ; \end{aligned}$$

42d $$\begin{aligned} B_{ij}^+=\frac{w_{ij}}{2} \left[ V_{ij}-\sqrt{\Delta _{ij} } \coth \left( \frac{\ell _{ij}\sqrt{\Delta _{ij} } }{2 D_{ij}}\right) \right] ; \end{aligned}$$

42e $$\begin{aligned} \omega _{ij}^- \!=\! \frac{w_{ij}f_{0,ij} }{2 K_{ij}}\left[ \sqrt{\Delta _{ij} } \frac{\exp \left( {{\sqrt{\Delta _{ij} } \ell _{ij}}/{D_{ij}}}\right) -2 \exp \left( {{\ell _{ij} \left( \sqrt{\Delta _{ij} }-V_{ij}\right) }/{2 D_{ij}}}\right) +1 }{\exp \left( {{\sqrt{\Delta _{ij} } \ell _{ij}}/{D_{ij}}}\right) -1}-V_{ij} \right] ; \nonumber \\ \end{aligned}$$

42f $$\begin{aligned} \omega _{ij}^+ \!=\! -\frac{w_{ij}f_{0,ij} }{2 K_{ij}}\left[ \sqrt{\Delta _{ij} }\,\frac{\exp \left( {{\sqrt{\Delta _{ij} } \ell _{ij}}/{D_{ij}}}\right) -2 \exp \left( {{\ell _{ij} \left( \sqrt{\Delta _{ij} }+V_{ij}\right) }/{2 D_{ij}}}\right) +1 }{\exp \left( {{\sqrt{\Delta _{ij} } \ell _{ij}}/{D_{ij}}}\right) -1}+V_{ij} \right] ;\nonumber \\ \end{aligned}$$

42g $$\begin{aligned} M_{ij} \!=\! -\frac{w_{ij} }{2 K_{ij}}\left[ \sqrt{\Delta _{ij} }\frac{\exp \left( {{\sqrt{\Delta _{ij}} \ell _{ij}}/{D_{ij}}}\right) -2 \exp \left( {{\ell _{ij} \left( \sqrt{\Delta _{ij} }+V_{ij}\right) }/{2 D_{ij}}}\right) +1 }{\exp \left( {{\sqrt{\Delta _{ij} } \ell _{ij}}/{D_{ij}}}\right) -1}+V_{ij} \right] ;\nonumber \\ \end{aligned}$$

42h $$\begin{aligned} N_{ij} \!=\! -\frac{w_{ij}}{2K_{ij}}\left[ \sqrt{\Delta _{ij} } \frac{\exp \left( {{\sqrt{\Delta _{ij} } \ell _{ij}}/{D_{ij}}}\right) -2 \exp \left( {{\ell _{ij} \left( \sqrt{\Delta _{ij} }-V_{ij}\right) }/{2 D_{ij}}}\right) +1 }{\exp \left( {{\sqrt{\Delta _{ij} } \ell _{ij}}/{D_{ij}}}\right) -1 }-V_{ij} \right] ;\nonumber \\ \end{aligned}$$

with $$\Delta _{ij} {:}{=}{V_{ij}^2 - 4D_{ij}K_{ij}}$$.

To build intuition, we consider a minimal network made of two identical nodes connected via two identical but oppositely oriented edges. The Laplacian tensor for this network is a $2\times 2$ matrix that can be computed easily using (19a, 42). The spectrum of this matrix characterises the scaling of the Laplacian w.r.t. to the various parameters, in particular the edge length $\ell$:

43 $$\begin{aligned} {{\,\textrm{Sp}\,}}\left( {\textbf{L}}\right) =&\left\{ - w \sqrt{V^2 - 4 K D} \left( \cosh \left( \frac{V\ell }{2 D}\right) +\cosh \left( \frac{\ell \sqrt{V^2-4 K D}}{2 D}\right) \right) {{\,\textrm{csch}\,}}\left( \frac{\ell \sqrt{V^2-4 K D}}{2 D}\right) , \nonumber \right. \\&\left. w \sqrt{V^2-4 K D} \left( \cosh \left( \frac{V\ell }{2 D}\right) -\cosh \left( \frac{\ell \sqrt{V^2 - 4 K D}}{2 D}\right) \right) {{\,\textrm{csch}\,}}\left( \frac{\ell \sqrt{V^2 - 4 K D}}{2 D}\right) \right\} . \end{aligned}$$

Note that, in the absence of a linear source along the edges ($K=0$), we recover the standard property that the Laplacian has zero as an eigenvalue. For short edges (that is, for $\ell \ll D/V$), we find the approximation

44 $$\begin{aligned} {{\,\textrm{Sp}\,}}\left( {\textbf{L}}\right) \approx \left\{ -4 Dw\ell ^{-1},\, K w \ell \right\} . \end{aligned}$$

As can be seen, this limit case supports the choice of the so-called *ballistic scaling* (Putra et al. 2021) where the edge weights are taken proportional to $\ell ^{-1}$. Conversely, it challenges the alternative quadratic scaling in $\ell ^{-2}$ which has been proposed based on the analogy with finite differences (Thompson et al. 2020). While previous studies have imposed these scalings constitutively into the Laplacian operator, our systematic approach derives the appropriate scaling directly from first principles and provides a means to assess their domain of validity. Moreover, it shows strong agreement with empirical data (Chaggar et al. 2025).

For long edges ($\ell \gg D/V$) and $K<0$, we have

45 $$\begin{aligned} {{\,\textrm{Sp}\,}}\left( {\textbf{L}}\right) \approx \left\{ - w \sqrt{V^2 - 4 KD} ,\, - w \sqrt{V^2 - 4 KD} \right\} , \end{aligned}$$

i.e., the dependency on edge length vanishes. Note that the eigenvalues are non-positive, unlike those of the traditional graph Laplacian which are non-negative. This is due to the sign convention chosen for the Laplacian, with a + instead of a − in front of $\textbf{L}$ in (1).

Finally, one can see that $\textbf{L}$ is a so-called *Metzler matrix*, meaning that its off-diagonal coefficients are all non-negative:

46 $$\begin{aligned} L_{ij} = A^+_{ji} - {B}^-_{ij} = \sqrt{\Delta _{ij}} w_{ij} \cosh \left( \frac{V_{ij} \ell _{ij}}{2 D_{ij}}\right) {{\,\textrm{csch}\,}}\left( \frac{\sqrt{\Delta _{ij}} \ell _{ij}}{2 D_{ij}}\right) \ge 0, \quad \forall i \ne j. \end{aligned}$$

Excluding for now the additional effect of the source terms $\hat{\mathcal {G}_i}$, this property ensures forward invariance of the positive orthant under the flow, that is, given initial conditions $\rho _i(0)\ge 0$ at $t=0$, the solution remains positive $\rho _i(t)\ge 0$ at $t>0$. This result extends directly to any source term generating a flow field which does not leave the positive orthant, i.e. such that $\mathcal {G}_i \ge 0$ at $\rho _i=0$ with $\rho _j\ge 0$ for all *j*. Indeed, the contribution of such an additional source term will not reverse the orientation of the overall flow field at the boundary of the positive orthant. This remark applies to the logistic-type growth term considered in Sect. 6, since the logistic function is positive near $0^+$.

### Diffusion-dominated case

It is interesting to examine the purely diffusive case with no reaction or advection. Here the solution established in Sect. 3 must be replaced by

47 $$\begin{aligned} x_{ij}(s) = \frac{s}{\ell _{ij}} \left( {\rho _j - \rho _i}\right) + \rho _i, \end{aligned}$$

with the fluxes at the boundaries given by

48 $$\begin{aligned} J_{ij}^- = J_{ij}^+ = \frac{D_{ij}\left( {\rho _i - \rho _j}\right) }{\ell _{ij}}. \end{aligned}$$

This solution yields the effective Laplacian (17, 19)

49 $$\begin{aligned} L_{ij} = \frac{D_{ij}w_{ji}}{\ell _{ji}} + \frac{D_{ji}w_{ij}}{\ell _{ij}} - \delta _{ij} \sum _{k=1}^N \left( {\frac{D_{ik}w_{ik}}{\ell _{ik}} + \frac{D_{ki}w_{ki}}{\ell _{ki}}}\right) . \end{aligned}$$

Strikingly, the previous expression corresponds exactly to the standard form of the Laplacian with ballistic weighting used by previous authors; see Putra et al. (2021). From the mass carried by an edge

50 $$\begin{aligned} m_{ij} = \frac{1}{2} \ell _{ij}w_{ij} \left( {\rho _i + \rho _j}\right) , \end{aligned}$$

we further obtain

51 $$\begin{aligned} M_{ij} = N_{ij} = \frac{\ell _{ij}w_{ij}}{2} . \end{aligned}$$

This justifies mechanistically the usage of network models based on the Laplacian which are appropriate to capture diffusion within the network edges, provided that the Laplacian is properly weighted.

## Application to the Fisher-Kolmogorov-Petrovsky-Piskunov equation

As an example, we examine the case of the Fisher-Kolmogorov-Petrovsky-Piskunov (henceforth FKPP) equation, which, among others, has been used as a minimal model for replication and diffusion of proteins across the brain connectome in neurodegenerative diseases (Fornari et al. 2019).

### General problem

The FKPP model is introduced through the logistic reaction term at the nodes:

52 $$\begin{aligned} \mathcal {G} (\rho ) = r^*\rho \left( {1-\epsilon ^* \rho }\right) . \end{aligned}$$

where $$r^* $$ and $$\epsilon ^*$$ are constants which measure the growth rate and saturation in the nodes, respectively (with the asterisk indicating nodal parameters). Along the edges, we posit (4)

53 $$\begin{aligned} - D x_{ij}'' + V x_{ij}' = r {x_{ij}} \left( {1-\epsilon x_{ij} }\right) , \end{aligned}$$

where we have assumed that *r*, $\epsilon$, *D*, *V* and *K* are uniform across all edges for simplicity. Choosing $\sqrt{D/r}$ and 1/*r* as reference length and time units respectively, we derive

54 $$\begin{aligned} - x_{ij}'' + \beta x_{ij}' = {x_{ij}} \left( {1- \epsilon x_{ij}}\right) \end{aligned}$$

with $\beta {:}{=}V/\sqrt{Dr}$ a dimensionless parameter. We see immediately that, for this system, $f_0 = 0$ and $K =1$. Since $K>0$, the maximum principle discussed in Sect. 3 does not apply here and the solution may in principle be negative. For small $\beta <2$, the solvability condition (41) requires that $\ell < 2\pi / \sqrt{4 -\beta ^2}$. Near this threshold the linear approximation blows up and fails to provide an accurate approximation as it misses the nonlinear saturation effect. Under the previous assumption on $\ell$, the solution is a linear combination of $\rho _i$ and $\rho _j$ with positive coefficients for all $s \in \left[ 0,\ell \right]$; thus, it is positive along the edge if both $\rho _i$ and $\rho _j$ are positive. Conversely, past this threshold, the coefficient vector leaves the positive orthant and the solution may become non-positive, thus, unphysical. In the gentler case where $\beta \ge 2$ (e.g. when the growth rate *r* is small), $\sqrt{4 -\beta ^2}$ is imaginary and solvability is universally guaranteed by (41), and the solution is positive.

Using (42), we obtain the eight constant matrices

55a $$\begin{aligned} A_{ij}^- = \frac{w_{ij}}{2} \left[ \beta +\sqrt{\beta ^2 - 4} \coth \left( \frac{\ell _{ij}\sqrt{\beta ^2 - 4} }{2}\right) \right] ,\end{aligned}$$

55b $$\begin{aligned} A_{ij}^+ = \frac{w_{ij}\sqrt{\beta ^2 - 4}}{2} \exp \left( {\frac{\beta \ell _{ij}}{2}}\right) {{\,\textrm{csch}\,}}\left( \frac{\ell _{ij}\sqrt{\beta ^2-4}}{2} \right) ,\end{aligned}$$

55c $$\begin{aligned} B_{ij}^-= -\frac{w_{ij}\sqrt{\beta ^2 - 4}}{2} \exp \left( {-\frac{\beta \ell _{ij} }{2 }}\right) {{\,\textrm{csch}\,}}\left( \frac{\ell _{ij}\sqrt{\beta ^2 - 4} }{2 }\right) ,\end{aligned}$$

55d $$\begin{aligned} B_{ij}^+= \frac{w_{ij}}{2} \left[ \beta -\sqrt{\beta ^2 - 4} \coth \left( \frac{\ell _{ij}\sqrt{\beta ^2 - 4} }{2}\right) \right] ,\end{aligned}$$

55e $$\begin{aligned} M_{ij} = -\frac{w_{ij}}{2 }\left[ \sqrt{\beta ^2-4 }\frac{\exp \left( {\ell _{ij}\sqrt{\beta ^2-4}}\right) -2 \exp \left( {{\ell _{ij} \left( \sqrt{\beta ^2-4 }+\beta \right) }/2}\right) +1}{\exp \left( {\ell _{ij}\sqrt{\beta ^2-4}}\right) -1}+\beta \right] ,\end{aligned}$$

55f $$\begin{aligned} N_{ij}= -\frac{w_{ij}}{2}\left[ \sqrt{\beta ^2-4} \frac{\exp \left( {\ell _{ij}\sqrt{\beta ^2-4}}\right) -2 \exp \left( {{\ell _{ij} \left( \sqrt{\beta ^2-4}-\beta \right) }/2}\right) +1}{\exp \left( {{\ell _{ij}\sqrt{\beta ^2-4}}}\right) -1}-\beta \right] , \end{aligned}$$

55g $$\begin{aligned} \omega _{ij}^- = 0,\quad \omega _{ij}^+ = 0. \end{aligned}$$

The Laplacian then results explicitly from (19):

56a $$\begin{aligned} L_{ij}&= \frac{\sqrt{\beta ^2 - 4}}{2} \left[ w_{ji} \exp \left( {\frac{\beta \ell _{ji}}{2}}\right) {{\,\textrm{csch}\,}}\left( \frac{\ell _{ji}\sqrt{\beta ^2-4}}{2} \right) \right. \nonumber \\&\quad \left. + w_{ij} \exp \left( {-\frac{\beta \ell _{ij} }{2 }}\right) {{\,\textrm{csch}\,}}\left( \frac{\ell _{ij}\sqrt{\beta ^2 - 4} }{2 }\right) \right] \nonumber \\&\quad - \frac{\delta _{ij}}{2} \sum _{k=1}^N \left[ w_{ik} \left( {\sqrt{\beta ^2 - 4} \coth \left( \frac{\ell _{ik}\sqrt{\beta ^2 - 4} }{2}\right) +\beta }\right) \right. \nonumber \\&\quad \left. + w_{ki} \left( {\sqrt{\beta ^2 - 4} \coth \left( \frac{\ell _{ki}\sqrt{\beta ^2 - 4} }{2}\right) -\beta }\right) \right] , \end{aligned}$$

56b $$\begin{aligned} \Omega _i = 0. \end{aligned}$$

In the case where $\beta \rightarrow 0$, i.e. $V\ll \sqrt{Dr}$, (55e), (55f), (56) reduce to the remarkably simple formulae

57 $$\begin{aligned} \begin{gathered} L_{ij} = w_{ij} \csc \ell _{ij} + w_{ji} \csc {\ell _{ji}} - \delta _{ij} \sum _{k=1}^N \left( {w_{ik} \cot \ell _{ik} + w_{ki} \cot \ell _{ki} }\right) , \\ \Omega _{i} = 0,\quad M_{ij} = N_{ij} = w_{ij}\tan \left( {\ell _{ij}/2}\right) . \end{gathered} \end{aligned}$$

Overall, we obtain the following system

58 $$\begin{aligned} \left( {\mathcal {V}_i + \sum _{j=1}^N \left( {M_{ij} + N_{ji}}\right) }\right) \dot{\rho }_i = \mathcal {V}_i r^*_i \rho _i (1-\epsilon ^*_i\rho _i) + \sum _{j=1}^N L_{ij} \rho _j ,\quad \forall i \in \mathcal {V}. \end{aligned}$$

### Weakly-nonlinear solution

The linearised FKPP model presented previously misses the quadratic saturation effect ($\epsilon$) in (54). To make progress, it is useful to consider the case of a small saturation $\epsilon \ll 1$. Through regular asymptotic expansion carried out to $O(\epsilon )$, we obtain a closed-form solution which depends quadratically on the $\rho _i$ and $\rho _j$:

59 $$\begin{aligned} x_{ij}(s) = \textbf{h}_{ij}(s) \cdot \boldsymbol{\rho }_{ij} + \epsilon \boldsymbol{\rho } _{ij}^\top \textbf{H}_{ij}(s) \boldsymbol{\rho }_{ij} + O(\epsilon ^2), \end{aligned}$$

with $\boldsymbol{\rho }_{ij}=\left( {\rho _i,\rho _j}\right)$; and where $\textbf{h}_{ij}$ and $\textbf{H}_{ij}$ are respectively a vector and matrix function of *s*, for which we derive a rather involved explicit expression (not shown here). The flux follows from (3):

60 $$\begin{aligned} w_{ij}J_{ij}(s) = \textbf{u}_{ij}(s) \cdot \boldsymbol{\rho }_{ij} + \epsilon \boldsymbol{\rho }^\top _{ij} \textbf{U}_{ij}(s) \boldsymbol{\rho }_{ij} , \end{aligned}$$

with

61 $$\begin{aligned} \quad \textbf{u}_{ij}(s){:}{=}{w_{ij}\beta \textbf{h}_{ij}(s)-w_{ij}\textbf{h}'_{ij}(s)},\quad \textbf{U}_{ij} (s){:}{=}{w_{ij}\beta \textbf{H}_{ij}(s) - w_{ij}\textbf{H}'_{ij}(s)} . \end{aligned}$$

In particular, we have

62 $$\begin{aligned} w_{ij}J_{ij}^\pm = A_{ij}^\pm \rho _i + B^\pm _{ij} \rho _j + U_{ij11}^\pm \rho _i^2+U_{ij22}^\pm \rho _j^2 +\left( {U_{ij12}^\pm +U_{ij21}^\pm }\right) \rho _i \rho _j, \end{aligned}$$

with $(A_{ij}^-,B_{ij}^-)=\textbf{u}_{ij}(0)$ and $(A_{ij}^+,B_{ij}^+)=\textbf{u}_{ij}(\ell _{ij})$; and $\textbf{U}_{ij}^- = \textbf{U}_{ij}(0)$ and $\textbf{U}_{ij}^+ = \textbf{U}_{ij}(\ell _{ij})$. Similarly, the mass carried by an edge is given by

63 $$\begin{aligned} m_{ij} = \int _0^{\ell _{ij}} w_{ij}x_{ij}(s)\textrm{d}{s} = \boldsymbol{\mathfrak {h}}_{ij} \cdot \boldsymbol{\rho }_{ij} + \epsilon \boldsymbol{\rho }_{ij} ^\top \mathbf {\mathfrak {H}_{ij}} \boldsymbol{\rho }_{ij}, \end{aligned}$$

with

64 $$\begin{aligned} \boldsymbol{\mathfrak {h}}_{ij} {:}{=}{\int _0^{\ell _{ij}} w_{ij}\textbf{h}_{ij}(s)\textrm{d}{s}}=\left( {M_{ij},N_{ij}}\right) ,\quad \boldsymbol{\mathfrak {H}} {:}{=}\int _0^{\ell _{ij}} w_{ij}\textbf{H}_{ij}(s)\textrm{d}{s}. \end{aligned}$$

Thus, starting from (16), and combining the previous expression for all edges (*i*, *j*) we obtain a higher-order system

65 $$\begin{aligned}&\hat{\mathcal {V}}_i(\rho _1,\dots ,\rho _N) \dot{\rho }_i = {\mathcal {V}}_i \hat{\mathcal {G}_i} \left( {\rho _i}\right) + \sum _{j = 1 }^N \left( {L_{ij} + \epsilon \rho _i\mathfrak {U}_{ij} }\right) \rho _j + \epsilon \mathfrak {V}_{ij}\rho _j^2 , \end{aligned}$$

with

66a $$\begin{aligned} \hat{\mathcal {V}}_i&{:}{=}\mathcal {V}_i + \sum _{j=1}^N M_{ij} + N_{ji} + \epsilon \left[ \left( {\mathfrak {H}_{ji12} +\mathfrak {H}_{ji21} +2 \mathfrak {H}_{ij11} }\right) \rho _i\right. \nonumber \\&\quad \left. + \left( {\mathfrak {H}_{ij12} +\mathfrak {H}_{ij21} +2 \mathfrak {H}_{ji22} }\right) \rho _j\right] ; \end{aligned}$$

66b $$\begin{aligned} \hat{\mathcal {V}}_i\hat{\mathcal {G}_i}(\rho _i){:}{=}\mathcal {V}_i \mathcal {G}_i \left( {\rho _i}\right) + \epsilon \rho _i^2 \sum _{j = 1 }^N U_{ji11}^+-U_{ij22} ^- ; \end{aligned}$$

66c $$\begin{aligned} \mathfrak U_{ij} = {U_{ji12}^+ +U_{ji21}^+ - U_{ij12}^- -U_{ij21}^-}; \quad \mathfrak V_{ij}{:}{=}{U_{ji22}^+ - U_{ij11}^- }. \end{aligned}$$

Expression (65) corresponds to the previous system (21) augmented with nonlinear terms of order $\epsilon$.

### Comparison of the different models

In this section we compare the different models of the network FKPP system. For simplicity we focus on the case $\beta =0$. We solve the full nonlinear problem (54) numerically on every edge and update the node densities accordingly following a forward Euler integration scheme. For simplicity, instead of evaluating ${\partial {m_{ij}}}/{\partial {\rho _i}}$ and ${\partial {m_{ij}}}/{\partial {\rho _j}}$ in (16), which is computationally heavy, we use the formulae (57) as an approximation. This numerical solution is compared with the linearised solution associated with the effective Laplacian (58), the purely diffusive case (49, 51), and the weakly nonlinear solution (65).

**Fig. 3:**
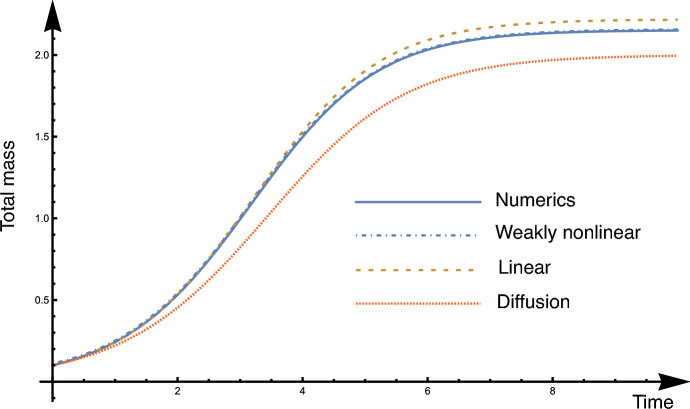
Example simulation of the FKPP process on a two-node network (total nodal mass). Here we compare the solution to the fully nonlinear FKPP model (54) computed numerically; the linearised version with effective Laplacian (57); the weakly nonlinear problem (65); and the purely diffusive problem (49, 51). Parameters: $\epsilon =0.25$, $\epsilon ^*=1$, $r^*=1$, $\ell =1$, $w=0.1$, $\beta =0$, $\mathcal {V}=1$.

An example simulation for a two-node network is shown in Fig. 3. Here the network densities are initialised with $\rho _0=0.1$ and $\rho _1=0$. As can be seen, for moderate saturation $\epsilon =0.25$ and edge volumes $\ell w = 0.1$, the effective Laplacian model provides a good approximation of the dynamics. The weakly nonlinear model further improves this approximation and the two curves (for the total nodal mass) are indistinguishable.

### Application to neurodegeneration: progression on the brain connectome

As a proof of concept, we briefly consider the example of toxic protein propagation in the context of Alzheimer’s disease. Misfolded proteins such as tau or $\alpha$-synuclein spread prion-like along axonal bundles, where they act as seeds that catalyze further misfolding, leading to exponential growth and eventual saturation within each brain region. By coarse-graining the brain into nodes representing anatomical regions connected through the connectome, the continuum FKPP model (Weickenmeier et al. 2018) reduces to a network version in which the graph Laplacian governs inter-regional transport while the nonlinear term captures local aggregation (Fornari et al. 2019). This network FKPP approach has been shown to reproduce key spatiotemporal patterns of disease progression, including Braak staging in Alzheimer’s disease, by predicting sequential regional invasion from primary seeds (Putra et al. 2021). Its parsimony and biological interpretability make it a valuable minimal model for investigating the interplay of transport, growth, and saturation in proteinopathies, and for generating clinically relevant predictions about disease staging and progression. Most studies using the network FKPP model use a graph Laplacian built from the weighted adjacency matrix. We demonstrate qualitatively how we can reproduce the dynamics of such systems based on our multiscale approach.

**Fig. 4:**
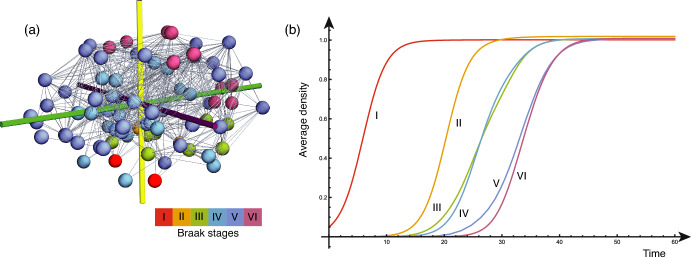
(**a**) The 83-node brain connectome and the Braak regions. (**b**) Example simulation of the spread of toxic proteins across the brain connectome, qualitatively in agreement with the Braak staging.

The 83-node connectome shown in Fig. 4(a) is constructed by partitioning the brain into $N=83$ anatomically defined regions of interest (nodes) based on a standard brain atlas, and representing the structural connections between them as edges. The connectivity is derived from diffusion tensor magnetic resonance imaging (DTI) tractography data from 418 healthy subjects of the Human Connectome Project, aggregated into the Budapest Reference Connectome v3.0 (Daducci et al. 2012). On the edges, we consider the advection-free FKPP model with $\beta =0$ (58, 57). Initially, there are no toxic proteins on the network ($\rho _i=0$) except in the entorrhinal cortex (shown in red in Fig. 4), seeded with a small initial amount of proteins. This initial state is unstable and leads to a cascading propagation across the entire network (parameters adapted from Putra et al. 2021).

Figure 4(b) shows the progression of the toxic load across the network, focusing on the six Braak regions, used to classify the progression of the disease, for which the average densities of toxic proteins across the region volumes are shown. As can be seen, the predicted staging of the disease is in good qualitative agreement with the Braak staging in this example. Further exploration of the parameter space can be performed following Putra et al. (2021).

## Concluding remarks

Using multi-compartment network models to represent spreading processes such as pathogen propagation across regions provides critical simplifications and mathematical insight, while drastically reducing computational costs, especially compared to continuum models (e.g., Weickenmeier et al. 2018). These models rely on a linear operator—typically the standard graph Laplacian—which accounts for the exchanges between the nodes of the network. Yet, the details of this operator have been mostly phenomenological and ad hoc (Putra et al. 2021). Using reaction-advection-diffusion as the paradigm process by which mass moves along the edges, we derived a multiscale theory where the fluxes between the nodes result directly from the solution of a differential equation along the edges. Assuming that densities along the edges remain small, linearisation yields an explicit form for the linear transport operator—the effective Laplacian. This operator reflects explicitly the physical quantities governing the transport process, as well as the geometry and topology of the network. Introducing the dynamics along the edges allows for the precise definition of an advection operator between nodes by accounting for the topology of the graph, thus providing a general method for constructing an active-transport process on the network (Benzi et al. 2025).

An important concern raised in this paper is the requirement that the transport operator respect mass conservation (Putra et al. 2021). Of course, most physical and biological systems of interest typically do not strictly conserve mass. In neurodegenerative diseases such as Alzheimer’s disease, for instance—the scenario motivating our work—proteins continually degrade, enter, and exit the system through multiple mechanisms, such as clearance and exocytosis. Nevertheless, from a conceptual perspective, it is important to ensure that *any* mass sources and sinks are properly modelled as separate contributions, so they appear deliberately as modelling choices, rather than as unintended side effects of a poorly chosen transport operator.

The method described in this paper further provides a theoretical basis to study the correct Laplacian weighting (Brennan and Goriely 2025). Typically, the diffusion scaling of the graph Laplacian (one over length squared) is justified as the discretisation of the diffusion operator (Thompson et al. 2020), whereas the ballistic scaling (one over length) assumes ballistic rather than Brownian dynamics in the edges. Yet, there is little systematic or mechanistic analysis undertaken to justify these scalings properly. Our approach provides a justification for the length-independent weighting (Abdelnour et al. 2014; Raj et al. 2012, 2015), which appears when the lengths of the edges are large (45) and in the presence of a strong active transport ($V^2>4DK$); and the ballistic weighting (Fornari et al. 2019, 2020; Putra et al. 2021) for short edges (44), or when the dynamics is dominated by diffusive effects (49). In contrast, the quadratic weighting—one over the edge length squared—which arises by canonical discretisation of the continuous Laplacian, is not captured by our method. Indeed, diffusion on a network is physically distinct from diffusion on a continuum domain, and the former may not be seen as a discretisation of the latter unless one consider a fine grid of nodes discretising the continuum, which is radically different from the idea of network evolution where large distances are bridged by edges.

The main limitation of our method is its reliance upon the assumption of linearity of the chemical reaction along the edges, precluding simple quadratic, or higher order reactions along the edges, nonlinear advection or non-Fickian diffusion. However, as illustrated in the case of the FKPP equation (Sect. 6.2), it is possible to expand the model asymptotically to include small higher order terms. Note also that the reactions at the level of the nodes can still be arbitrarily nonlinear, as no particular hypothesis need be made regarding the functions $\mathcal {G}_{i}^\mu$. Indeed, the goal of our model is not to include detailed nonlinear mechanisms such as nonlinear modulation of tau-protein advection along axons in Alzheimer’s disease (Bertsch et al. 2025; Tora et al. 2025), but rather to exhibit dominant behaviours and key scaling relationships. This approach then provides insight into the first order behaviour of the system, allowing for exhibiting the dominant behaviour of more complex nonlinear models solved only numerically.

Our framework bridges microscale transport mechanisms with macroscale network models, thereby providing the long-missing justification for the use of graph Laplacians in a wide range of applications. Starting from first principles, our work provides a theoretical foundation for an important class of models and paves the way for their systematic application across biological, physical, and technological networks.

## Data Availability

Wolfram Mathematica notebooks are available upon request.
